# Intraoral appliance treatment modulates inflammatory markers and oxidative damage in elderly with sleep apnea

**DOI:** 10.1590/1807-3107bor-2024.vol38.0084

**Published:** 2024-12-09

**Authors:** Alessandra Peres, João Carlos Fraga da Rosa, Joane Severo Ribeiro, Sofia de Lima Silva, Cristiane Bündchen, Gilson Pires Dornelles, Vania Fontanella

**Affiliations:** a Universidade Federal de Ciências da Saúde de Porto Alegre - UFCSPA, Laboratory of Cellular and Molecular Immunology, Porto Alegre, RS, Brazil.; b Moinhos de Vento Hospital, Porto Alegre, RS, Brazil.; c Universidade Federal do Rio Grande do Sul, School of Dentistry, Department of Surgery and Orthopedics, Porto Alegre, RS, Brazil.

**Keywords:** Sleep Apnea, Obstructive, Mandibular Advancement, Biomarkers, Aged

## Abstract

Obstructive sleep apnea (OSA) causes intermittent hypoxia, increased production of reactive oxygen species, and inflammation, which may elevate morbidity and mortality from cardiovascular disease. The objective of the present study was to investigate the effect of the intraoral appliance (IOA) as a treatment for OSA when it comes to the modulation of inflammatory markers and oxidative damage in elderly individuals. This "before and after" clinical trial included 9 patients diagnosed with OSA recruited from a multicenter randomized clinical trial study that evaluated the treatment with IOA for 60 days. Demographic and anthropometric variables, apnea and hypopnea index (AHI), and oxygen desaturation index (ODI) were collected by type III polysomnography, Epworth Sleepiness Scale, and inflammatory and oxidative damage markers (interleukin 6 (1L-6); tumor necrosis factor α (TNF-α); interleukin 10 (IL-10); thiobarbituric acid reactive substances (TBARS); total thiols; advanced oxidation protein products (AOPP), and nitric oxide (NO)). Shapiro-Wilk test, paired t-test and Pearson's correlation tests were used to analyze the results, respectively (α=0.05). The sample had a mean age of 71.86 ± 4.63 years, the majority were women (55.55%), and had a significant reduction in AHI (p=0.003), ODI (p=0.038), IL-10 (p=0.0001), AOPP (p=0.038) and TBARS levels (p=0.0001). There was a significant correlation between IL-10 and NO (r=0.855) and between TBARS and IL-6 (r=0.669), both after treatment. This study demonstrated that treating elderly patients with OSA using an IOA for 60 days reduces oxidative damage through the modulation of AOPP and TBARS.

## Introduction

Obstructive sleep apnea (OSA) is a chronic, multifactorial disease that generally affects airflow in the upper airways and has a high prevalence in elderly individuals.^1^ Its management must be multidisciplinary since the treatment depends on the severity of the symptoms, the magnitude of the clinical complications, and the etiology of upper airway obstruction. The CPAP (Continuous Positive Airway Pressure) is the gold standard treatment for moderate-to-severe OSA. However, its cost is very high, and treatment adherence can sometimes be unsatisfactory.^2^

Therefore, the evaluation of alternative methods for the treatment of OSA is crucial. Intraoral appliances (IOA) are a good alternative since they are already well established in clinical treatment and recognized by the American Association of Sleep Medicine.^3^ They offer some advantages, such as being a non-invasive and reversible therapy, it is accepted by most patients, it has low cost and is easy to manufacture.^3,4^ The IOA is a device that stabilizes the jaw in a protruding position to keep the airway open during sleep. A customized IOA is fabricated on models of the maxillary and mandibular arches and can be titratable, allowing different degrees of mandibular protrusion, or non-titratable. The non-customized IOA is mostly prefabricated and partially modified to adapt to the patient's arches.^4^

The effectiveness of the IOA is generally related to the degree of mandibular advancement, reducing the collapsibility of the upper airway.^5^

Craniofacial and soft tissue characteristics have a significant influence on airway constriction.^5^ Factors related to this syndrome include obesity, mandibular deficiency, muscle hypotonia, excessive fat on the palate, tongue, and pharynx, and elongated soft palate, among others.^6^

OSA is characterized by repeted episodes of interruption and reduction of airflow during sleep, accompanied by a decrease in blood oxygen saturation, despite continuous respiratory effort, causing changes in molecular and physiological pathways.^7^ Constant chronic inflammatory processe and imbalance of oxidative stress are the main factors contributing to the cardiovascular morbidity and mortality in patients with OSA.^8,9^ There is evidence that pro-inflammatory cytokines such as interleukin-1β (IL-1β), interleukin-6 (IL-6), and tumor necrosis factor α (TNF α) are involved in the physiology of sleep regulation and the relationship of these inflammatory markers with OSA. At the resolution of an immune response, the anti-inflammatory process is triggered to limit immune activation, and IL-10 is one of the critical anti-inflammatory cytokines with an essential role in controlling inflammation.^10^ In addition, airway collapse in OSA causes mechanical injury leading to increased release of reactive oxygen species (ROS) and reduced physiological antioxidant capacity, generating increased levels of oxidative stress compared to non-apneic individuals.^11^

It was demonstrated that using CPAP decreases inflammatory markers, such as C-reactive protein levels and interleukin 6 (IL-6) by monocytes.^12^ Furthermore, lipid peroxidation is an important oxidative stress marker since lipids are easily oxidized, and some studies^13^ demonstrated that lipid peroxidation levels were reduced using the gold standard treatment.

It is essential to understand the mechanisms related to the treatment of OSA with IOA and the associated modulation of the immune system. Thus, the present study proposed to evaluate the effect of IOA treatment on inflammatory markers and oxidative damage in elderly patients with OSA.

## Methods

### Study design and ethics

This research is a before-and-after study of an independent sample recruited from a controlled, double-blind, multicenter, randomized clinical trial (RCT) approved by the National Research Ethics Committee under Opinion No. 2.581/776, registered at the Brazilian Registry of Clinical Trials (ReBEC), and all experimental procedures were performed according to the Declaration of Helsinki. All participants were informed about the study and signed the informed consent.

### Recruitment and procedures

**Figure 1:**
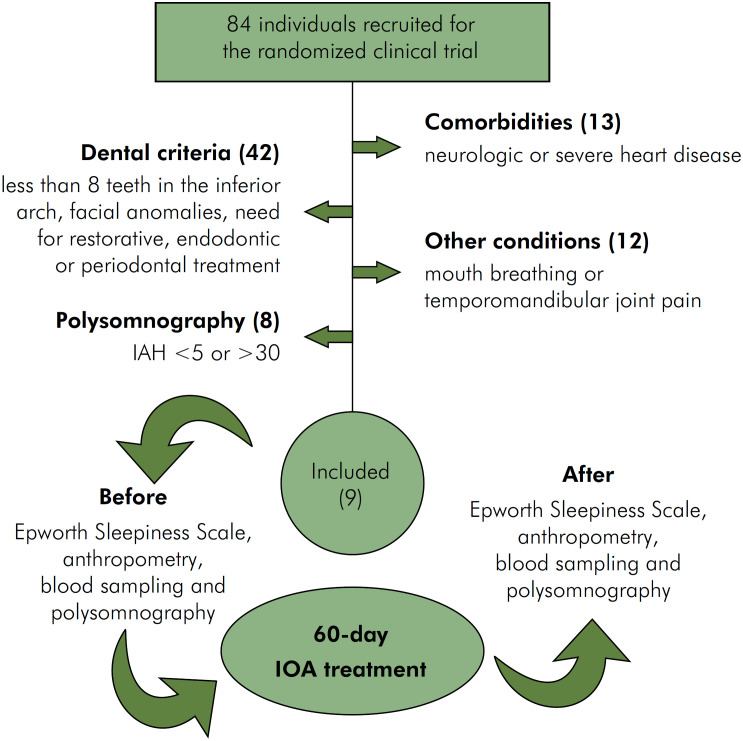
Flowchart of study participants from recruitment to 60 days of follow-up.

Participants recruited from public health services were entered sequentially and according to their availability. Of the 84 participants recruited to the RCT, only 9 met the eligibility criteria for this study. The exclusion criteria were comorbidities such as severe heart or neurological disease, fewer than eight teeth in the lower arch, facial anomalies or neoplasms, need for restorative, endodontic or periodontal treatment, mouth breathing, temporomandibular joint pain, and recent change in eating habits or physical activities. All recruited individuals who met these criteria (n=17) underwent baseline type III polysomnography and those who presented apnea-hypopnea index (AHI) < 5 or > 30 were also excluded. Participants also underwent anthropometric evaluation, Epworth Sleepiness Scale (ESS) evaluation, and blood sampling before treatment with IOA. After 60 days using a custom-made and titratable IOA, participants were evaluated again with the same examination protocol as at baseline. Throughout the study, participants were accompanied by a dentist and a physical therapist who performed all examinations. The methodological design of the study is summarized in Figure 1.

### Measurements at baseline and follow-up (60 days)

All clinical measurements were made by a physiotherapist. Demographic data (gender, age) were recorded, and the ESS questionnaire was self-administered to measure daytime sleepiness. Anthropometric measurements of neck circumference, waist and hip circumference (cm), height (cm), and weight (kg) were taken during the same appointment under standardized conditions, always using the same equipment and by the same examiner. From the body mass index (BMI) calculation, participants were categorized into average weight (BMI ≤ 24.9), overweight (≥ 25.0 and ≤ 29.9), and obese (≥ 30.0).^14^

### Parameters for obtaining the indices of apnea, hypopnea (AHI) and oxygen desaturation (ODI)

To perform type III polysomnography with a portable monitor (ApneaLink^®^, ResMed Corporation, Poway, USA), the participants were instructed on how to use the device and place the sensors (nasal cannula, pulse oximeter, and respiratory effort belt) for subsequent use without supervision at home. According to the AASM criteria^3^, the results were analyzed by a trained and calibrated technician blinded to all clinical and laboratory tests. The criterion to obtain the oxygen desaturation index (ODI) was a 3% drop in the baseline obtained automatically by the equipment. OSA presence and severity were classified according to the number of events per hour as normal (AHI < 5), mild (AHI 5 to ≤15), moderate (AHI 15 to < 30), and severe (AHI ≥ 30).^3^

### Blood sample collection and analysis method

A single examiner performed blood sample collection. All samples were collected in the morning with the instruction not to perform any physical activity before the blood collection. The blood samples were used for the analysis of inflammatory markers interleukin 6 (IL-6), tumor necrosis factor alpha (TNF-α) and interleukin 10 (IL-10), nitric oxide (NO), and oxidative damage markers thiobarbituric acid (TBARS), total thiols, and advanced protein oxidation products (AOPP). Blood (8 mL) was collected from the antecubital vein in heparinized tubes and tubes without anticoagulant, before (baseline) and after 60 days of treatment with IOA. The tubes were centrifuged (1,000 g) for 10 minutes. Aliquots were frozen in microtubes kept at −80º C until analyses. The concentrations of IL-6, IL-10, and TNF-alpha were determined by enzyme-linked immunosorbent (ELISA) assay using a commercial kit (PeproTech, Cranbury, NJ, USA). The coefficient of variation between assays was < 7.5%.

TBARS concentration in plasma was determined by spectrophotometry according to a previously described protocol^15^, and data were expressed as nmol/mL. Nitrite concentrations were analyzed according to the established methodology^16^, and results were expressed in μM/L. The reading wavelength was 540 nm. AOPP analysis was performed in plasma according to a previously established protocol^17^. Total thiols were evaluated by reacting 25 μL of plasma with 450 μL of phosphate buffer (0.2 M, pH 8.0) and 25 μL of 5,5’-dithiobis- (2-nitrobenzoic acid) (DTNB, 10 mM, Sigma-Aldrich^®^, St. Louis, USA) for 30 min in the dark. After this incubation period, 200 μL were transferred to microplates, and reading by spectrophotometry was performed at 412 nm wavelength in a Biochrom EZ Read 400 plate reader (Harvard Bioscience, Holliston, USA).^18^

### Statistical Analysis

The data were analyzed using the statistical program SPSS version 22.0 (IBM, Armonk, USA). The normality of the data was verified using the Shapiro-Wilk test, and the characteristics of the individuals and the variables studied were submitted to descriptive statistics. Data were log-transformed when distribution was not normal. The paired t-test was used to compare variables before and after IOA treatment, and Pearson's correlation test was used to verify correlation between variables. The significance level adopted was 5% for all analyses.

## Results

Nine participants completed the treatment. Demographic and clinical characteristics are described in Table 1. The mean age of the participants was 71.86 ± 4.63 years, and 55.55% were female. Table 2 shows that there was a significant improvement in AHI (p = 0.003) and ODI (p = 0.038).

**Table 1:** Characteristics of the sample recruited for intraoral appliance treatment (n = 9).

Sex (%)	Female : male
	5:4 (55.55:44.45)
	Mean (minimum - maximum)
Age (years)	71.86 (67–79)
Body mass index baseline (kg/m^2^)	28.06 (24.4–32)
Body mass index after (kg/m^2^)	28.28 (25–32)
Neck circumference baseline (cm)	35.83 (33–38)
Neck circumference after (cm)	35.83 (33–38)
Waist circumference baseline (cm)	95.39 (86–112)
Waist circumference after (cm)	95.66 (87–112)
Hip circumference baseline (cm)	106.77 (96–128)
Hip circumference after (cm)	106.88 (96–128)
Epworth Sleepiness Scale baseline	3.77 (1–10)
Epworth Sleepiness Scale after	3.89 (1–6)

**Table 2:** Distribution of polysomnography parameters before (baseline) and after 60 days of treatment with an intraoral appliance.

Parameter	Baseline	After 60 days	Difference from the medians	p-value*
AHI	16.72 (5.9–26.8)	10.36 (3.9–16.4)	-6.36	0.003
ODI	14.22 (4.3–24.8)	8.8 (4.3–12.6)	-5.42	0.038

AHI: apnea and hypopnea index; ODI: oxygen desaturation index;

* T test.

**Figure 2:**
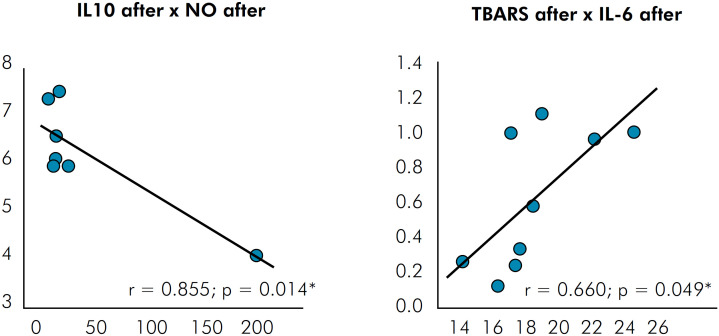
Correlations between inflammatory and oxidative damage markers. NO (nitric oxide); IL-6 (interleukin-6); IL-10 (interleukin 10); TBARS (thiobarbituric acid).

In the comparison between inflammatory and oxidative damage markers at baseline and after treatment with IOA, there was a significant decrease in IL-10, AOPP, and TBARS (p < 0.05) (Table 3). However, there was a significant correlation between IL-10 and nitric oxide after treatment (r = 0.855) and between TBARS and IL-6 after treatment (r = 0.669) (Figure 2).

**Table 3:** Inflammatory markers and oxidative damage levels before (baseline) and after 60 days of treatment with intraoral appliance.

Markers	Baseline	After 60 days	p-value*
IL-6 (pg/mL)	18.20 ± 3.16	19.00 ± 2.92	0.551
IL-10 (pg/mL)	6.78 ± 2.75	5.97 ± 1.17	0.0001*
TNF-α (pg/mL)	23.07 ± 8.00	23.66 ± 8.11	0.421
NO (μM/L)	59.61 ± 55.12	38.91 ± 64.77	0.199
Thiols (nmol/mL)	260.84 ± 65.83	169.25 ± 99.40	0.066
AOPP (µmol/mL)	200.76 ± 16.78	164.48 ± 45.40	0.038*
TBARS (mmol/mL)	9.20 ± 2.80	6.08 ± 5.04	0.0001*

IL-6: interleukin-6; IL-10: interleukin 10; TNF-α: tumor necrosis factor alpha; NO: nitric oxide; AOPP: protein oxidation products; TBARS: thiobarbituric acid;

* T test.

**Figure 3:**
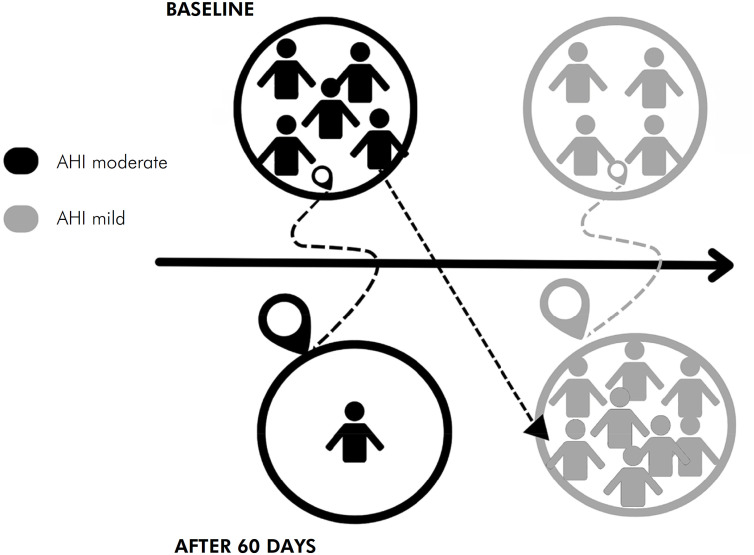
Graphical representation of the apnea degree shift after treatment with IOA.

Figure 3 shows the change of AHI category after treatment.

## Discussion

In the present study, the effect of IOA treatment reducing AHI and ODI was observed. In addition, a reduction in markers of oxidative damage was observed in elderly subjects after 60 days of treatment.

As in our study, Hedberge et al.^13^ found a significant reduction of > 50% in AHI from baseline to 90 days of treatment with mandibular advancement IOA. In another study^19^, treatment with IOA for 180 days resulted in significant apnea reduction, indicating that this treatment is an effective alternative in patients with moderate-to-severe OSA who refused CPAP. A study comparing the effect of IOA treatment between young and elderly patients over five years observed no age-related difference in outcomes. However, elderly must be monitored for possible dental adverse effects more frequently since long-term treatment is more efficacious.^20-23^

In the present study, TBARS, a marker of oxidative damage, decreased significantly after 60 days, contrary to the results of Gupta et al.^21^ after a 2-year treatment follow-up. However, the study population was adult subjects aged 41 years in average. Peres et al.^24^ analyzed 402 patients and verified an association between the marker 8-isoprostane and OSA severity. In the comparison between CPAP and IOA treatment, there was no decrease in TBARS; however, catalase levels were reduced the group treated with IOA.^25^ In addition to TBARS, a reduction in AOPP levels was observed, indicating a reduction in cell membrane damage.

It is known that apnea leads to significant sleep disturbances and that these interruptions can favor changes in systemic cytokine concentrations. Sleep deprivation leads to the imbalance of pro-inflammatory cytokines such as IL-1β, TNF-α, and IL-6.^10^ In the present study, the concentration of pro-inflammatory cytokines (IL-6 and TNF-α) did not change after 60 days of treatment with IOA, but IL-10 levles, an anti-inflammatory cytokine, were reduced. These findings corroborate the results of Hedberg et al.,^13^ even using a 90-day treatment. In a multicenter study^26^ with 391 patients with OSA, CPAP treatment for six months did not affect TNF-α, IL-6, and IL-10 levles compared to the control group. In contrast, in studies with IOA treatment for 180 days or more, a significant reduction in IL-β and TNF-α was found compared to the control group.^19^ Another study verified a modulation in IL-10 levels, but not in IL-6 levels, 1 year after treatment with IOA compared to baseline.^23^

As limitations of the study, the chronic inflammation process normally observed in the elderly population may have interfered with the modulation of inflammatory markers. In addition, the diet of the participants was not assessed.^27^ Furthermore, the small sample size makes it impossible to extrapolate the results to the general elderly population, affecting this study's external validity. It is noteworthy that in addition to the expected presence of comorbidities, 50% of the recruited individuals had dental conditions that did not allow the treatment of OSA with IOA, impairing the use of this therapeutic modality. On the other hand, some strengths should be highlighted, such as the measurement of the outcomes being standardized and performed by a single trained examiner blinded to the research groups. Moreover, the investigation of various inflammatory and oxidative damage markers without any loss to follow up at the end of 60 days contributed to understanding the IOA mechanism of action on inflammatory markers and oxidative damage.

## Conclusion

The treatment of apneic elderly patients with IOA for a minimum period of 60 days has a positive effect on the modulation of TBARS and AOPP, which reflects on the reduction of oxidative damage.

## References

[1] Peppard PE, Young T, Barnet JH, Palta M, Hagen EW, Hla KM (2013). Increased prevalence of sleep-disordered breathing in adults. Am J Epidemiol.

[2] Sawyer AM, Gooneratne NS, Marcus CL, Ofer D, Richards KC, Weaver TE (2011). A systematic review of CPAP adherence across age groups: clinical and empiric insights for developing CPAP adherence interventions. Sleep Med Rev.

[3] Berry RB, Brooks R, Gamaldo C, Harding SM, Lloyd RM, Quan SF (2017). AASM scoring manual updates for 2017 (Version 2.4). J Clin Sleep Med.

[4] Ramar K, Dort LC, Katz SG, Lettieri CJ, Harrod CG, Thomas SM (2015). Clinical practice guideline for the treatment of obstructive sleep apnea and snoring with oral appliance therapy: an update for 2015. J Clin Sleep Med.

[5] Banhiran W, Assanasen P, Metheetrairut C, Chotinaiwattarakul W (2013). Health-related quality of life in Thai patients with obstructive sleep disordered breathing. J Med Assoc Thai.

[6] Genta PR, Schorr F, Eckert DJ, Gebrim E, Kayamori F, Moriya HT (2014). Upper airway collapsibility is associated with obesity and hyoid position. Sleep.

[7] Lévy P, Kohler M, McNicholas WT, Barbé F, McEvoy RD, Somers VK (2015). Obstructive sleep apnoea syndrome. Nat Rev Dis Primers.

[8] Ryan S, Taylor CT, McNicholas WT (2009). Systemic inflammation: a key factor in the pathogenesis of cardiovascular complications in obstructive sleep apnoea syndrome?. Postgrad Med J.

[9] Orrù G, Storari M, Scano A, Piras V, Taibi R, Viscuso D (2020). Obstructive Sleep Apnea, oxidative stress, inflammation and endothelial dysfunction-An overview of predictive laboratory biomarkers. Eur Rev Med Pharmacol Sci.

[10] Irwin MR (2019). Sleep and inflammation: partners in sickness and in health. Nat Rev Immunol.

[11] Passali D, Corallo G, Yaremchuk S, Longini M, Proietti F, Passali GC (2015). Oxidative stress in patients with obstructive sleep apnoea syndrome. Acta Otorhinolaryngol Ital.

[12] Karamanlı H, Özol D, Ugur KS, Yıldırım Z, Armutçu F, Bozkurt B (2014). Influence of CPAP treatment on airway and systemic inflammation in OSAS patients. Sleep Breath.

[13] Hedberg P, Nohlert E, Tegelberg Å (2021). Effects of oral appliance treatment on inflammatory biomarkers in obstructive sleep apnea: A randomised controlled trial. J Sleep Res.

[14] World Health Organization. WHO Consultation on Obesity (2012). Obesity: preventing and managing the global epidemic. Report of a World Health Organization Consultation.

[15] Ohkawa H, Ohishi N, Yagi K (1979). Assay for lipid peroxides in animal tissues by thiobarbituric acid reaction. Anal Biochem.

[16] Miranda KM, Espey MG, Wink DA (2001). A rapid, simple spectrophotometric method for simultaneous detection of nitrate and nitrite. Nitric Oxide.

[17] Witko-Sarsat V, Friedlander M, Capeillère-Blandin C, Nguyen-Khoa T, Nguyen AT, Zingraff J (1996). Advanced oxidation protein products as a novel marker of oxidative stress in uremia. Kidney Int.

[18] Ellman GL (1959). Tissue sulfhydryl groups. Arch Biochem Biophys.

[19] Fernández-Julián E, Pérez-Carbonell T, Marco R, Pellicer V, Rodriguez-Borja E, Marco J (2018). Impact of an oral appliance on obstructive sleep apnea severity, quality of life, and biomarkers. Laryngoscope.

[20] Marklund M, Franklin KA (2015). Treatment of elderly patients with snoring and obstructive sleep apnea using a mandibular advancement device. Sleep Breath.

[21] Gupta A, Tripathi A, Sharma P (2017). The long-term effects of mandibular advancement splint on cardiovascular fitness and psychomotor performance in patients with mild to moderate obstructive sleep apnea: a prospective study. Sleep Breath.

[22] Galic T, Bozic J, Ivkovic N, Gunjaca G, Ticinovic TK, Dogas Z (2016). Effects of mandibular advancement device treatment on arterial stiffness and glucose metabolism in patients with mild to moderate obstructive sleep apnea: a prospective 1 year study. Sleep Breath.

[23] Niżankowska-Jędrzejczyk A, Almeida FR, Lowe AA, Kania A, Nastałek P, Mejza F (2014). Modulation of inflammatory and hemostatic markers in obstructive sleep apnea patients treated with mandibular advancement splints: a parallel, controlled trial. J Clin Sleep Med.

[24] Peres BU, Allen AJ, Shah A, Fox N, Laher I, Almeida F (2020). Obstructive sleep apnea and circulating biomarkers of oxidative stress: a cross-sectional study. Antioxidants.

[25] Dal-Fabbro C, Garbuio S, D'Almeida V, Cintra FD, Tufik S, Bittencourt L (2014). Mandibular advancement device and CPAP upon cardiovascular parameters in OSA. Sleep Breath.

[26] Stradling JR, Craig SE, Kohler M, Nicoll D, Ayers L, Nunn AJ (2015). Markers of inflammation: data from the MOSAIC randomised trial of CPAP for minimally symptomatic OSA. Thorax.

[27] Zuo L, Prather ER, Stetskiv M, Garrison DE, Meade JR, Peace TI (2019). Inflammaging and oxidative stress in human diseases: from molecular mechanisms to novel treatments. Int J Mol Sci.

